# A One-year Follow-up Study on Predictors of Temporary Leaves and Drop-outs among Students at a Women's Junior College

**DOI:** 10.2188/jea.18.26

**Published:** 2008-02-28

**Authors:** Hideko Murai, Takeo Nakayama

**Affiliations:** 1 Department of Integrated Life, Nutrition Course, Osaka Seikei Junior College; 2 Department of Health Informatics, Kyoto University School of Public Health

**Keywords:** Students, Follow-Up Studies, Student Dropout, Mental Health, School Health Services

## Abstract

**Background:**

In Japan, the temporary leave and drop-out rate of university or junior college students has been increasing in recent years, and many cases have been attributed to psychological problems. To establish a mental health support system for entering students, we conducted a questionnaire and follow-up survey, and explored predictors of temporary leaves and drop-outs among junior college women.

**Methods:**

Our sample consisted of 485 first-year female students attending a junior college in Osaka, Japan. Between 1998 and 2002, the following factors were assessed: lifestyle, college life, subjective well-being measured by the General Well-Being Schedule (GWBS), self-esteem, and emotional support network. A follow-up survey was conducted during 1 year.

**Results:**

Thirty-seven women, who had taken temporary leaves or had dropped out during the first year, showed unfavorable responses to lifestyle, college life and/or subjective well-being compared with other students. No differences in self-esteem and emotional support network were found between the two groups. A multiple regression analysis showed that non-existence of interesting club activity, smoking, and low level of life satisfaction and emotional stability measured by the GWBS were predictors of temporary leaves and drop-outs.

**Conclusion:**

It may be possible to determine which students are at risk for taking temporary leaves or dropping out based on their psychological state and lifestyle at the time of enrollment in college. More support is needed to continue the students at school who are at high risk for taking temporary leaves or dropping out.

## INTRODUCTION

Japan has one of the most advanced education systems in the world: more than 47% of high school students enter university or junior college after graduation.^1^ However, according to the Comprehensive Survey of Living Conditions of the People on Health and Welfare, the increasing number of advancing students experience anxiety and stress regarding schoolwork, test taking, and advancing to the next grade.^2^ For students who have just taken an entrance examination for college, an irregular lifestyle, feelings of relief, physical and psychological stress, and an inability to adapt socially are common. These unfavorable conditions and psychological instability are thought to result in students taking temporary leaves or dropping out after having entered university or junior college. According to a survey conducted at a national university between 1981 and 1987, approximately 1% of enrolled students either took a temporary leave or dropped out, and 40% of each group left for psychological reasons, including apathy and depression.^3^ In addition, in a follow-up study, Nakamura et al^4^ found that students who repeated a grade or dropped out had shown unfavorable responses relating to a decline of motivation or feelings of inadequacy at the time of university enrollment.

In Japan, the temporary leave and drop-out rate of university and junior college students is not published in detail, but available data indicate that the rate is much lower than those of Western nations.^5^ It appears that some Japanese students take temporary leaves or drop out to change professional direction or consider other universities.^6^ In fact, the temporary leave and drop-out rate in Japan has been gradually increasing in recent years.^6^ Moreover, previous studies have reported that many students who took temporary leaves or dropped out did so because of psychological problem.^3^^,^^4^^,^^6^

There is deep-rooted idea that universities or junior colleges are educational institutions where students learn by their own volition in Japan. As such, the need for institutional coping strategies and guidance for students has not yet been fully recognized. However, once a student enters university or junior college, the school has an obligation to prevent "burnout" and provide that student with the necessary professional knowledge to play an active role in society. Institutions must not only provide education, but also help students maintain their physical and psychological health.

To date, studies on the problem of temporary leaves or drop-outs in universities and junior colleges have primarily consisted of observational studies^3^^,^^6^^-^^11^ with a limited number of follow-up studies.^4^^,^^12^^,^^13^ We conducted a questionnaire survey during an orientation week at a women's junior college located in Osaka Prefecture in Japan to help establish a mental health support system for first-year students. This survey began in April 1998 and was designed to examine the state and changes of students' mental health and associated factors. We conducted a follow-up study on first-year students to test the hypothesis that factors related to students' lifestyle and psychological instability influenced the prevalence of temporary leaves and drop-outs.

## METHODS

### Sample and Study Design

The survey sample consisted of female students enrolled in the nutrition course at a women's junior college in Osaka Prefecture from 1998 to 2002. This was a retrospective cohort study using existing data, which consisted of information taken from a self-administered questionnaire survey upon enrollment and information on temporary leaves and drop-outs during the first year obtained from school records. Using a data set combining the former with the latter, we explored predictors of temporary leaves and drop-outs among junior college women.

### Self-administered Questionnaire Survey and Contents

In Japan, school begins in April. At that time, the authors conducted a self-administered questionnaire survey to determine the female students' mental health at the first class of the semester during the orientation period. The survey was administered each April (for 5 years) in the morning. All participants provided consent after being informed that their cooperation was voluntary, that all personal information would remain private and only be used for the present study's statistical analysis, and that their answers would not affect their course evaluation.

Self-administered questionnaires were identical for all the 5 years. The following items were included in data analysis. Results for each item were obtained by having participants select one answer from a multiple-choice question during the previous month. A total of 11 lifestyle items contained multiple-choice questions with 2 to 6 possible responses. The lifestyle items comprised health awareness (1 item); stress awareness (3 items: "amount of stress", "stress tolerance", and "positive thinking"); health behavior (6 items: "sleeping duration", "eating breakfast", "nutritional balance", "smoking status", "alcohol consumption", and "frequency of athletic activity") and commuting (1 item) (Table 1). Nine items were used to assess college life; these items consisted of multiple-choice questions with 3 to 5 possible responses. The items were "expectations of college life", "satisfaction with college entered", "decision about where one has entered college", "existence of interesting club activity", "ability to make friends", "good relationships with faculty members", "confidence regarding college life", "objectives for college life", and "desired career" (Table 1). To measure mental health, we used three subscales of the Japanese General Well-Being Schedule (GWBS). The original GWBS is a self-administered questionnaire that contains 18 items consisting of 6 subscales developed by Dupuy^14^ to assess psychological well-being and distress. A Japanese version with 17 items was developed consisting of 3 subscales ("depression", "health concerns", and "life satisfaction and emotional stability").^15^ The range of scores on the 3 subscales was 0 to 30 for "depression", 0 to 30 for "health concerns", and 0 to 45 for "life satisfaction and emotional stability". The total score on the GWBS was 105, with higher scores representing better health. Self-esteem and emotional support network were measured as psychosocial factors that influenced students' mental health. Some previous studies have shown that self-esteem^16^^,^^17^ or social support levels^18^^,^^19^ are related to adaptation to the new environment for students enrolled in college and influence mental health or the quality of life at college. Thus, we measured self-esteem and the presence of an emotional support network to determine student's social support. We evaluated self-esteem using the Japanese version of Rosenberg's Self-esteem Scale^20^ to assess students' self-esteem. Developed by Munakata,^21^ this self-rating scale consists of 10 items scored on a 4-point scale (1=strongly disagree; 2=disagree; 3= agree; 4=strongly agree). The total score ranges from 10 to 40, with higher scores indicating greater self-esteem. To assess the emotional support network instead of social support, 3 items related to "one's emotional support network" were adopted from the Emotional Support Network Awareness Scale.^22^ These 3 items evaluated whether subjects had any of following types of supports available: "someone who helps you feel relaxed and who you are relieved to meet", "someone who agrees with and supports your behaviors or ideas", and "someone who you can confide in about your feelings and secrets". Responses to each item were "available" (1 point) or "not available" (0 point), with larger scores (possible score range, 0 to 3) indicating a larger emotional support network.

**Table 1.** Lifestyle and college life items and their categories.

Item	Good (Desirable or positive response)	Poor (Undesirable or negative response)
Lifestyle		
Health awareness	Very good Good	Poor Very poor

Amount of stress	None at all A little A moderate amount	Much Very much

Stress tolerance	High Fairly high Moderate	Poor Very poor

Positive thinking	Positive thoughts Somewhat positive thoughts Neither negative nor positive thoughts	Somewhat negative thoughts Negative thoughts

Sleeping duration	≧ 6 hrs./night	< 6 hrs./night

Eating breakfast	Everyday Everyday, but simply	3-4 times/week Never

Nutritional balance	Always Occasionally	Seldom Never

Smoking status	Non-smoker Non-smoker over 1 year Stopped smoking during the past year	Occasionally Everyday

Alcohol consumption	Non-drinker Past-drinker 2-3 times/month	1-3 times/week 4-5 times/week Everyday

Frequency of athletic activity	≧ 3 times/week 1-2 times/week	Seldom

Commuting from home	Yes	No

College Life		
Expectations of college life	Extremely high expectations Fairly high expectations Moderately high expectations	Low expectations No expectations

Satisfaction with college entered	Extremely satisfied Fairly satisfied Moderately satisfied	Somewhat dissatisfied Dissatisfied

Decision about where one has entered college	Decided by myself Finally decided by myself after hearing opinions	Not decided by myself

Existence of interesting club activity	Many interesting club activities Moderate number of interesting club activities Neither a high nor low number	Few interesting club activities No interesting club activities

Ability to make friends	I think so I somewhat think so	Can't say I somewhat do not think so I do not think so

Good relationships with faculty menbers	I think so I somewhat think so	Can't say I somewhat do not think so I do not think so

Confidence regarding college life	I think so I somewhat think so	Can't say I somewhat do not think so I do not think so

Objectives for college life	Many objectives Moderate number of objectives Neither a high nor low number	Few objectives No objectives

Desired career	Strongly desired career Moderately desired career Neither high nor low desire in a specific	Low desire in a specific career No desired career

### Information on Temporary Leaves and Drop-outs

In the present study sample, requests for temporary leaves or drop-outs from students were handled as follows: First, the faculty talked to the student who wanted to leave school, and on a case-by-case basis, advised the student to consider leaving completely or returning to school after taking a temporary leave. There are no fixed criteria to categorize the different rationales for leaving school because the faculty member in charge of dealing with temporary leaves and drop-outs changes each year. The faculty member in charge of the nutrition program asked the faculty member in charge of dealing with temporary leaves and drop-outs to provide the reason that student took a temporary leave or dropped out, and after it was approved, the faculty member in charge of the nutrition course was the final judge on the reasons for temporary leaves or drop-outs. Then, the college officer moved forward with the proper procedure for dealing with temporary leaves or drop-outs. Based on information obtained, we were able to categorize the reasons into 5 categories based on his findings: "problems with schoolwork" (including "mistaken enrollment" [attending this school because their parent's pushed them or they were rejected from their first-choice school] or "a change of professional direction"), "financial difficulty at home", "health problems", "marriage", and "unknown" in cases where the reason was not revealed (Table 2).

**Table 2.** Survey sample and reasons for temporary leaves and/or drop-outs during the first year of college.

Survey year	Sample size	No. of subjects (%)		

		Temporary leave (n=4)	Drop-out (n=33)	Total (n=37)
1998	86		3 ( 4)	3 ( 4)
1999	106		9 ( 9)	9 ( 9)
2000	104	1 ( 1)	4 ( 4)	5 ( 5)
2001	96	3 ( 3)	10 (10)	13 (13)
2002	93		7 ( 8)	7 ( 8)

Reasons for temporary leave and/or drop-out	Problems with schoolwork*	1 ( 3)	21 (57)	22 (60)
	Financial difficulty at home		6 (16)	6 (16)
	Health problems	3 ( 8)	1 ( 3)	4 (11)
	Marriage		1 ( 3)	1 ( 3)
	Unknown		4 (11)	4 (11)

*: Mistaken enrollment, change of professional direction

"Mistaken enrollment" means attending this school because their parent's pushed them or they were rejected from their first-choice school.

The authors obtained information on temporary leaves and drop-outs during the first year using the following methods. First, the purpose of the study was presented to the faculty member who oversees the nutrition program. That person is responsible for maintaining information on temporary leaves and drop-outs. We then received permission to use the data in our survey. The authors temporarily kept the survey data with each student's name and research identification number in a personal computer. We requested that the faculty member match the students' name and research identification number on the survey data with the information on temporary leaves and drop-outs and then delete all student names to protect confidentiality. The faculty member then forwarded the anonymous information (i.e. all names were deleted) to us. We conducted the present study using this data. After the follow-up survey ended, we obtained the information from the faculty member in charge of the nutrition that one student who had taken a temporary leave had re-enrolled and the rest dropped out. Thus, the authors judged that most students who took temporary leaves ended up dropping out completely. Therefore, students who took temporary leaves or dropped out during the first year were categorized as a single group, and those who remained in school for the second year were categorized as another group.

We focused on first-year students for three reasons. First, in this present survey, 86% of all temporary leaves and drop-outs (43 students) during 2 years were first-year students (37 students). Thus, we considered it urgent to address the problem of temporary leaves or drop-outs during the first year. Second, because entering school is a stressful life event itself,^23^ it is a factor involved with creating an unstable mental health state and influencing the quality of college life.^24^^,^^25^ In addition, previous analysis of 1998 and 1999 survey revealed that both mental health state and responses to college life among first-year students tended to be poorer than those among second-year students, indicating that many first-year students succeeded in adapting to college life after 1 year.^26^ Hence, we considered that mental health state and college life among first-year students were likely related to temporary leaves and drop-outs. Third, it was impossible to analyze data during 2 years because minimal data among second-year students were available (6 students) and drop-outs occurred sporadically (5 students in 1999 and 1 student in 2002). Although addition data collection would be helpful, this data collection would take a long time because of the limited number of temporary leaves or drop-outs during the periods we studied. Therefore, in the present study, we used existing data to focus on factors related to temporary leaves and drop-outs only during the first year. Further research is necessary to analyze factors related to temporary leaves and drop-outs by grade and to determine changes from the first year to the second year.

### Ethical Consideration

The methodology of this study conducted using existing data was approved by the Ethics Review Board of the Japan Epidemiological Association on February 24, 2005.

### Statistical Analysis

Students' lifestyle was evaluated as follows: based on previous studies,^27^^-^^31^ a desirable choice is "good" and an undesirable one is "poor" for each the 11 items on lifestyle. In the present study, a desirable "sleeping duration" was revised as "6 or more hours" because in our preliminary survey, 82% of students responded that they slept for 6 or more hours (including 45% of students who slept for 6 hours). The responses to the 9 items on college life showed a single-peak distribution that inclined toward the right and left. Based on previous studies,^3^^,^^4^^,^^6^^,^^29^ the responses to the 9 items were categorized into 2 groups: a positive choice is "good" and a negative choice is "poor". For both the lifestyle and college life items, a score of "0" was assigned to an undesirable or negative choice and a score of "1" was assigned to a desirable or positive choice (Table 1). We used the Mantel-Haenszel test to distinguish differences in lifestyle and college life responses between students who remained in school and those who took temporary leaves or dropped out. We used the Mann-Whitney U test to compare GWBS, self-esteem, and emotional support network scores between these two groups for each year. Next, we assessed which factors affected temporary leaves and drop-outs. With temporary leaves and drop-outs as dependent variables, we conducted a multiple logistic regression analysis of significant items obtained in the Mantel-Haenszel test for lifestyle and college life, the 3 subscales of the GWBS, self-esteem, and emotional support network. Statistical analyses were conducted on SPSS^®^ 13.0J for Windows. The existence of multicollinearity between independent variables on lifestyle and college life was examined by Pearson's correlation coefficient. Statistical significance for all tests was defined as p<0.05.

## RESULTS

The survey sample consisted of female students enrolled in the nutrition course at a women's junior college in Osaka Prefecture from 1998 to 2002. Total enrollment was 87 in 1998, 106 in 1999, 105 in 2000, 97 in 2001, and 96 in 2002. Except for 6 students -- 1 student who did not fill out the questionnaire, 4 students who entered school following a periods of employment or work in the home, and 1 student who re-enrolled after leaving school -- all 485 students responded to the questionnaire (Table 2). A total of 474 subjects were 18 years old, 10 were 19 years old, and 1 was 20 years old, (mean age, 18.0 years, standard deviation = 0.2).

Table 2 showed temporary leaves and drop-outs by survey year. Four students took temporary leaves and 33 students dropped out during the first year after enrollment, and 448 students remained in school for the second year. Thus, a total of 37 students (total incidence: 8%) either took a temporary leave or dropped out during the present survey; 33 of those 37 students (89%) dropped out. The primary reasons for temporary leaves and drop-outs were "problems with schoolwork" (n=22, 60%) followed by "financial difficulty at home" (n=6, 16%), and "health problems" (n=4, 11%) (Table 2) .

After adjusting for survey year, we analyzed the difference in response trends on lifestyle and college life between students who remained in school and those who took temporary leaves and dropped out. Table 3 shows the number of subjects who chose "poor" responses among each group. Temporary leaves and drop-outs were found to be significantly related to "smoking" (p=0.002), "lack of breakfast" (p=0.011), and "short sleeping duration (<6 hrs/night) " (p=0.032). For other items, including "health awareness", "amount of stress", "nutritional balance", and "frequency of athletic activity", there were more "poor" responses among students who took temporary leaves and dropped out than among those who remained in school in spite of the lack of significance. Analyses of college life found a significant difference between students who remained in school and those who took temporary leaves and dropped out for the following 5 reasons: "existence of interesting club activity" (p=0.010), "decision about where one has entered college" (p=0.013), "satisfaction with college entered" (p=0.022), "expectations of college life" (p=0.032), and "objectives for college life" (p=0.035).

**Table 3.** Rates of poor respondants regarding lifestyle and college life at the time of enrollment during all survey years: Students who remained in school compared with those who took temporary leaves and dropped out during the first year of college.

Item	Total		1998		1999		2000		2001		2002		
	
	Temporary leave and drop-out (n = 37)	Remained (n=448)	Temporary leave and drop-out (n=3)	Remained (n=83)	Temporary leave and drop-out (n=9)	Remained (n=97)	Temporary leave and drop-out (n=5)	Remained (n=99)	Temporary leave and drop-out (n=13)	Remained (n=83)	Temporary leave and drop-out (n=7)	Remained (n=86)	p value*
Lifestyle, No. of subjects who gave poor responses (%)													
Health awareness	10 ( 27)	79 ( 18)		10 ( 12)	2 ( 22)	18 ( 19)	1 ( 20)	14 ( 14)	3 ( 23)	14 ( 17)	4 ( 57)	23 ( 27)	0.292
Amount of stress	18 ( 49)	174 ( 39)	1 ( 33)	35 ( 42)	3 ( 33)	45 ( 46)	3 ( 60)	39 ( 39)	6 ( 46)	30 ( 36)	5 ( 71)	25 ( 29)	0.287
Stress tolerance	12 ( 32)	158 ( 35)	2 ( 67)	30 ( 36)	1 ( 11)	36 ( 37)	3 ( 60)	39 ( 39)	5 ( 39)	23 ( 28)	1 ( 14)	30 ( 35)	0.972
Positive thinking	18 ( 49)	232 ( 52)	2 ( 67)	39 ( 47)	2 ( 22)	54 ( 56)	3 ( 60)	55 ( 56)	7 ( 54)	44 ( 53)	4 ( 57)	40 ( 47)	0.812
Sleeping duration	12 ( 32)	77 ( 17)	1 ( 33)	13 ( 15)	2 ( 22)	12 ( 12)	1 ( 20)	23 ( 23)	4 ( 31)	14 ( 17)	4 ( 57)	15 ( 17)	0.032
Eating breakfast	7 ( 19)	28 ( 6)		4 ( 5)	1 ( 11)	6 ( 6)	1 ( 20)	7 ( 7)	2 ( 15)	4 ( 5)	3 ( 43)	7 ( 8)	0.011
Nutritional balance	25 ( 68)	251 ( 56)	3 (100)	48 ( 58)	6 ( 66)	47 ( 49)	2 ( 40)	62 ( 63)	8 ( 62)	40 ( 48)	6 ( 86)	54 ( 63)	0.151
Smoking status	12 ( 32)	54 ( 12)		3 ( 4)	3 ( 33)	15 ( 16)	2 ( 40)	13 ( 13)	4 ( 31)	8 ( 10)	3 ( 43)	15 ( 17)	0.002
Alcohol consumption	3 ( 8)	32 ( 7)		5 ( 6)		5 ( 5)	1 ( 20)	12 ( 12)	1 ( 8)	5 ( 6)	1 ( 14)	5 ( 6)	0.993
Frequency of athletic activity	33 ( 89)	349 ( 78)	3 (100)	59 ( 71)	9 (100)	74 ( 76)	5 (100)	84 ( 85)	10 ( 77)	66 ( 80)	6 ( 86)	66 ( 77)	0.164
Commuting from home	9 ( 24)	82 ( 18)	2 ( 67)	16 ( 19)	3 ( 33)	22 ( 23)	1 ( 20)	15 ( 15)	1 ( 8)	18 ( 22)	2 ( 29)	11 ( 13)	0.531

College Life, No. of subjects who gave poor responses (%)													
Expectations of college life	9 ( 24)	53 ( 12)		20 ( 24)	2 ( 22)	12 ( 12)		7 ( 7)	3 ( 23)	8 ( 10)	4 ( 57)	6 ( 7)	0.032
Satisfaction with college entered	10 ( 27)	59 ( 13)	1 ( 33)	18 ( 22)	1 ( 11)	19 ( 20)		11 ( 11)	5 ( 39)	6 ( 7)	3 ( 43)	5 ( 6)	0.022
Decision about where one has entered college	7 ( 19)	28 ( 6)	1 ( 33)	9 ( 11)	1 ( 11)	5 ( 5)		5 ( 5)	3 ( 23)	7 ( 8)	2 ( 29)	2 ( 2)	0.013
Existence of interesting club activity	34 ( 92)	315 ( 70)	2 ( 67)	59 ( 71)	9 (100)	70 ( 72)	4 ( 80)	70 ( 71)	13 (100)	58 ( 70)	6 ( 86)	58 ( 67)	0.010
Ability to make friends	6 ( 16)	68 ( 15)		22 ( 27)	1 ( 11)	13 ( 13)		10 ( 10)	2 ( 15)	12 ( 15)	3 ( 43)	11 ( 13)	0.956
Good relationships with faculty menbers	21 ( 57)	197 ( 44)	1 ( 33)	35 ( 42)	5 ( 56)	46 ( 47)	4 ( 80)	52 ( 53)	7 ( 54)	34 ( 41)	4 ( 57)	30 ( 35)	0.153
Confidence regarding college life	12 ( 32)	100 ( 22)	1 ( 33)	28 ( 34)	1 ( 11)	25 ( 26)	2 ( 40)	22 ( 22)	4 ( 31)	13 ( 16)	4 ( 57)	12 ( 14)	0.127
Objectives for college life	9 ( 24)	51 ( 11)	1 ( 33)	12 ( 15)	2 ( 22)	13 ( 13)		9 ( 9)	3 ( 23)	7 ( 8)	3 ( 43)	10 ( 12)	0.035
Desired career	4 ( 11)	25 ( 6)	1 ( 33)	8 ( 10)	1 ( 11)	5 ( 5)	1 ( 20)	5 ( 5)		3 ( 4)	1 ( 14)	4 ( 5)	0.212

*: Mantel-Haenszel method adjusted for each survey year

Table 4 shows the average scores among each group per year for the GWBS, self-esteem, and emotional support network. We found that temporary leaves or drop-outs were significantly associated with the GWBS subscale's "life satisfaction and emotional stability" (p=0.017) and total score (p=0.021) in 2000; "depression" (p=0.038) in 2001, and "life satisfaction and emotional stability" (p=0.040), total score (p=0.038), and self-esteem (p=0.006) in 2002. As a whole, scores for students who took temporary leaves or dropped out were lower than those who remained in school.

**Table 4.** Scores for GWBS, self-esteem, and emotional support network by year of study: Students who remained in school compared with those who took temporary leaves and dropped out during the first year of college.

Survey Year	Item		Mean ± standard deviation			p value*

			Total (n=485)	Temporary leave and drop-out (n=37)	Remained (n=448)	
1998	No.		86	3	83	
	GWBS	Depression	20.6 ± 5.2	18.7 ± 3.8	20.7 ± 5.2	0.370
		Health concerns	14.0 ± 4.8	12.3 ± 3.8	14.1 ± 4.9	0.555
		Life satisfaction and emotional stability	20.9 ± 6.5	15.3 ± 1.5	21.1 ± 6.6	0.077
		Total score	55.6 ± 14.3	46.3 ± 5.9	55.9 ± 14.4	0.161
	Self-esteem		24.5 ± 4.4	24.0 ± 2.7	24.5 ± 4.5	0.723
	Emotional support network		2.7 ± 0.7	3.0 ± 0.0	2.7 ± 0.7	0.441

1999	No.		106	9	97	
	GWBS	Depression	21.7 ± 5.9	20.6 ± 5.1	21.8 ± 6.0	0.301
		Health concerns	14.5 ± 5.0	16.4 ± 3.5	14.4 ± 5.1	0.201
		Life satisfaction and emotional stability	22.7 ± 6.8	20.9 ± 4.7	22.9 ± 6.9	0.315
		Total score	58.9 ± 14.9	57.9 ± 10.7	59.0 ± 15.3	0.586
	Self-esteem		24.9 ± 4.9	26.9 ± 4.1	24.7 ± 5.0	0.211
	Emotional support network		2.8 ± 0.5	3.0 ± 0.0	2.8 ± 0.6	0.224

2000	No.		104	5	99	
	GWBS	Depression	22.0 ± 5.3	17.8 ± 6.2	22.2 ± 5.2	0.073
		Health concerns	15.8 ± 4.7	12.4 ± 3.0	16.0 ± 4.7	0.072
		Life satisfaction and emotional stability	23.3 ± 6.6	16.0 ± 5.5	23.7 ± 6.5	0.017
		Total score	61.1 ± 13.6	46.2 ± 13.3	61.9 ± 13.3	0.021
	Self-esteem		25.2 ± 4.9	22.8 ± 6.9	25.3 ± 4.8	0.406
	Emotional support network		2.9 ± 0.4	3.0 ± 0.0	2.9 ± 0.4	0.457

2001	No.		96	13	83	
	GWBS	Depression	21.1 ± 5.8	17.3 ± 7.6	21.7 ± 5.3	0.038
		Health concerns	14.9 ± 3.7	14.9 ± 3.9	14.9 ± 3.7	0.727
		Life satisfaction and emotional stability	20.9 ± 6.5	18.1 ± 7.6	21.4 ± 6.2	0.076
		Total score	57.0 ± 13.5	50.3 ± 15.1	58.0 ± 13.1	0.089
	Self-esteem		25.2 ± 4.7	25.1 ± 5.5	25.2 ± 4.6	0.851
	Emotional support network		2.8 ± 0.6	2.6 ± 0.8	2.9 ± 0.5	0.144

2002	No.		93	7	86	
	GWBS	Depression	21.8 ± 5.4	16.4 ± 10.1	22.3 ± 4.7	0.161
		Health concerns	15.0 ± 4.3	13.1 ± 4.8	15.1 ± 4.3	0.223
		Life satisfaction and emotional stability	22.1 ± 5.8	17.1 ± 6.7	22.6 ± 5.6	0.040
		Total score	58.9 ± 12.4	46.7 ± 18.5	59.9 ± 11.3	0.038
	Self-esteem		25.4 ± 4.3	20.7 ± 4.5	25.8 ± 4.1	0.006
	Emotional support network		2.9 ± 0.4	2.6 ± 1.1	3.0 ± 0.2	0.248

*: Mann-Whitney U test for each survey year

Table 5 shows the results of the multiple regression analysis. Items found to be significant were "existence of interesting club activity" (4.53, 95% confidence interval=1.31-15.66), "smoking status" (2.40, 95% confidence interval=1.04-5.54), and "life satisfaction and emotional stability (GWBS subscale)" (1.09, 95% confidence interval=1.01-1.18). Students who answered negatively on existence of interesting club activity were 4.5-times more likely to take temporary leaves or drop out; students who smoked were 2.4-times more likely to take temporary leaves or drop out; students who dropped 1 point on the GWBS subscale score (life satisfaction and emotional stability) were 1.1-times more likely to take temporary leaves or drop out. Pearson's correlation coefficient between independent variables on lifestyle and college life ranged from - 0.047 to 0.365; therefore the possibility of multicollinearity was considered small.

**Table 5.**
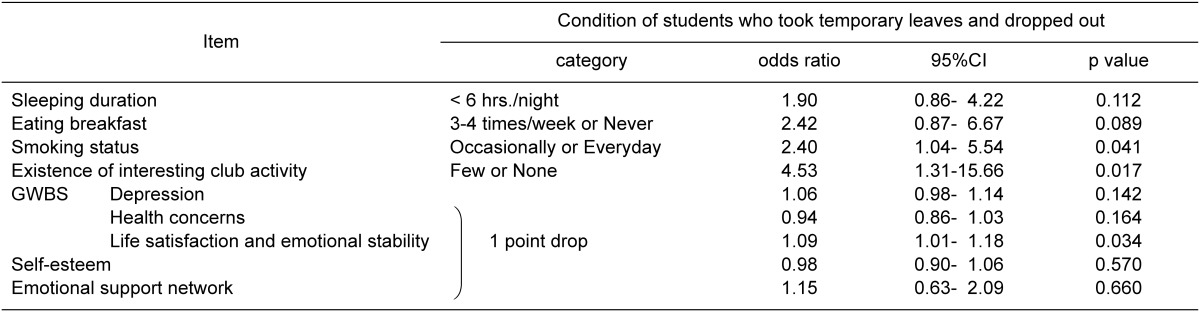
Relationship between taking temporary leaves and dropping out during the first year of college and lifestyle, college life, GWBS, self-esteem, and emotional support network. (Multiple logistic regression analysis [all variables])

CI: confidence interval

## DISCUSSION

We evaluated first-year college students to determine what factors lead to taking temporary leaves or dropping out. However, because this was a retrospective cohort study using existing data, some information may be lacking and results may not be sufficient to make generalizations. For example, our sample was limited to female students enrolled in a nutrition course at a women's junior college. The actual number of temporary leaves and drop-outs was very low and the follow-up was limited to 1 year. However, there are few follow-up studies on temporary leaves or drop-outs among first-year college students,^4^^,^^12^ and there have been no follow-up studies in which social, psychological, college life, and lifestyle factors helped predict the desire to leave school early. Therefore, even with these limitations, this study serves as the first follow-up study that examines factors predicting which students will request temporary leaves or drop-outs, and thus provides valuable information for further research.

The temporary leave and drop-out rate in our study was 8% for all survey years (37 out of 485 students). Because our study was limited to a single college and a single group, it is difficult to compare with other studies. However, though the survey method and the age of subjects are different, the temporary leave/drop-out rate was much higher than that seen in another study of female college students (temporary leave rate; 2% and drop-out rate; 1%).^6^ The reasons for temporary leaves and drop-outs in the other study were primarily related to problems with schoolwork, including poor academic performance and insufficient credits, and psychological problems, especially apathy.^6^ This was similar to the findings in our study. Several studies of the drop-out rates of medical and nursing students in Europe and America have been performed.^7^^,^^8^^,^^10^^,^^11^ These studies reported a drop-out rate of 4% to 19%, with the primary reasons for drop-outs being problems with academic performance and the onset of depressive symptoms from academic stress. Although the subjects and study type differ from our study, the primary reason for dropping out was also similar to that seen in our study.

The study subjects were in first-year students who entered a women's junior college. First-year students would feel relief and more relaxed after successfully completing an examination for junior college; however, some students moved away from home to live alone in an apartment (in the present study, approximately 20% students lived away from home), leading to additional stress. Likewise, lifestyles were irregular among most students. Previous cross-sectional studies reported that unhealthy lifestyles negatively influence students attitudes about school, affecting both attendance and academic performance.^28^^-^^31^ Kagawa et al^28^ found a correlation showing that students who skipped breakfast missed class more often and had poorer grades. Iijima et al^30^ found an association between days absent and regularity of diet, intake of breakfast, and sleeping duration. Trockel et al^31^ reported that later wake-up times among first-year students were associated with poor academic performance. In the present study, Mantel-Haenzel method revealed a significant relationship between an unhealthy lifestyle, especially "smoking", "lack of breakfast", and "short sleeping duration"(<6 hrs/night) and temporary leaves or drop-outs during the first year. Furthermore, we conducted a multiple logistic regression analysis and found that "smoking" was as independent predictor of temporary leaves and drop-outs but "lack of breakfast" and "short sleeping duration" were not. A cross-sectional study on smoking among women's university students found that an average of 16% of students smoke.^32^ This figure fluctuated among majors with more than 20% of students in the arts being smokers and less than 10% of students in domestic-economic majors, including nutrition, being smokers. In the present study, for all 5 years, 66 of 485 students (14%) responded that they had smoked during the previous month. This percentage appears to be high when compared with that among students (10%) in domestic-economic majors in the study done by Ohwada et al.^32^ Among the 66 students in the present study who had smoked within the previous month, 12 of them (18%) either took a temporary leave or dropped out during their first year. Because smoking is officially illegal for adolescents and most of our sample was under-age, it is possible that this figure underestimated the current incidence of smoking. Muramatsu^33^^,^^34^ reported that female university student smokers consumed more alcohol, used more make-up, had larger allowances, and went on holiday more often than non-smokers. Several other reports also found that smoking was significantly related to lower grades, decreased academic motivation, and other unhealthy lifestyle (e.g. greater consumption of fast food and drinking alcohol) among junior high and high school students.^35^^-^^38^ Our findings agree that smoking is an independent predictor for temporary leaves or drop-outs. Risk factors for a loss of academic motivation and, ultimately, for temporary leaves and drop-outs, are clearly related to an unhealthy lifestyle (e.g. smoking).

Students in the present study were enrolled in a 2-year course to become dietitians, which required them to take a series of mandatory courses to qualify for graduation. The required curriculum for dietitians is more intense compared with similar qualifications in the humanities. We surmise that students who were not satisfied with college and who had not solidified their goals had difficulty with the subject matter and began to experience frustration with schoolwork. These same students would then lose motivation and thus be inclined to skip classes, take temporary leaves, or drop out.^3^^,^^4^^,^^6^ According to Monden's cross-sectional study examining the relationship between college life among students at a women's junior college and the Cornell Medical Index, which measures the psychosomatic health of students, students who showed low levels of adaptation and satisfaction with college life were more likely to complain of psychological problems than the norm.^29^^,^^39^ This study also found that students with lower grades were more likely to fall into a depressive state. Studies in Western countries have shown similar results. Wagner et al^40^ tested for an association between psychological symptoms and the occurrence of a stressful event among junior high, high school, and college students and found that, among college students in particular, academic stress was strongly related to psychological symptoms. Last et al^41^ found that the drop-out rate among students in nursing schools was associated with factors of organizational stress and concern, including feelings of a lack of clinical skills, not being valued as a student nurse, and the unmet expectations of their academic program. Our findings showed that students who took temporary leaves and dropped out tended to respond negatively regarding "expectations of college life", "satisfaction for college entered", "decision of where one has entered college", and "objectives for college life". They also scored lower on the GWBS subscales of "depression" and "life satisfaction and emotional stability". Logistic regression analysis found a low score in "life satisfaction and emotional stability" to be a risk factor for temporary leaves or drop-outs. These findings are similar to a previous study in which an evaluation of mental health and college life were also related to temporary leaves and drop-outs in first-year students.^4^

Findings highlight that "non-existence of interesting club activity" was an independent factor for temporary leaves sand drop-outs. Previous studies had found that students belonging to a club activity showed less anxiety,^30^ were optimistic,^42^ and rarely become emotionally unstable.^43^ Club participation serves as an emotional support system, relieves students of stress, and allows them to maintain emotional stability. According to a survey conducted by the Cabinet Office, 72% of Japanese subjects between the ages of 18 and 24 years felt content "with friends and peers".^44^ In times of stress and concern, approximately 60% of individuals discussed their problems with "friends from school or one's community". More than 80% of first-year students in the present study were confident of their "ability to make friends". This suggests that first-year students would make friends in class and participate in club activities, which facilitated communication and allowed them to remain emotionally stable. However, non-existence of interesting club activities could correlate to an inability to make friends. This would then translate into a decrease in the student's satisfaction and expectations for college life, which, ultimately, brings about poor mental health.

First-year students may also experience academic, social, or financial problems in their new environment. Previous studies reported that high levels of social support from friends, family, or teachers moderated life stressors and loneliness, promoted adaptation to the new environment, and influenced mental health or quality of life at college,^18^^,^^19^ High self-esteem is maintained by establishing a good relationship with the people around the circumference and is thought to be important in maintaining good mental health.^16^^,^^17^ In the present study, respective scores for emotional support network instead of social support and self-esteem showed little disparity between students who took temporary leaves and dropped out and those who remained in school. For emotional support network, both groups scored an average of 2.8 of 3 points. For self-esteem, scores were similar to the national averages.^45^^,^^46^ This finding indicates that "interpersonal relationships" were unrelated to temporary leaves and drop-outs. Despite the fact that students who took temporary leaves and dropped out had a strong emotional support network and sufficient self-esteem, their mental health, as determined by the GWBS, was significantly lower. Further research is needed to determine how factors beyond interpersonal relationships, including "problems with schoolwork", "financial difficulty at home", and "health problems" affect mental health.

We conducted the present study to observe whether we could predict factors related to first-year students' temporary leaves or drop-outs. Results showed that "non-existence of interesting club activity", "smoking", and "low life satisfaction and emotional stability" measured by the GWBS were predictors of temporary leaves and drop-outs among first-year students.

In conclusion, it may be possible to determine which students are at risk for taking temporary leaves or dropping out based on their psychological state and lifestyle at the time of enrollment in college. Additional studies from various patient populations, as well as cross-cultural comparisons, are needed to help guide the development of a mental health support program for college students who are at high risk for taking a temporary leave or dropping out.
